# Effect of Supplementation with* n*-3 Fatty Acids Extracted from Microalgae on Inflammation Biomarkers from Two Different Strains of Mice

**DOI:** 10.1155/2018/4765358

**Published:** 2018-04-01

**Authors:** L. E. Gutiérrez-Pliego, B. E. Martínez-Carrillo, A. A. Reséndiz-Albor, I. M. Arciniega-Martínez, J. A. Escoto-Herrera, C. A. Rosales-Gómez, R. Valdés-Ramos

**Affiliations:** ^1^Laboratorio de Investigación en Nutrición, Facultad de Medicina, Universidad Autónoma del Estado de México, Paseo Tollocan y Venustiano Carranza s/n, Col. Universidad, 50180 Toluca, MEX, Mexico; ^2^Laboratorio de Inmunidad de Mucosas, Sección de Investigación y Posgrado, Escuela Superior de Medicina, Instituto Politécnico Nacional, Av. Plan de San Luis S/N, Colonia Casco de Santo Tomas, Miguel Hidalgo, 11350 Ciudad de México, Mexico

## Abstract

**Background:**

Diabetes mellitus is considered a chronic noncommunicable disease in which inflammation plays a main role in the progression of the disease and it is known that* n*-3 fatty acids have anti-inflammatory properties. One of the most recent approaches is the study of the fatty acids of microalgae as a substitute for fish oil and a source rich in fatty acids EPA and DHA.

**Objective:**

To analyze the effect of supplementation with* n*-3 fatty acids extracted from microalgae on the inflammatory markers from two different strains of mice.

**Methods:**

Mice of two strains, db/db and CD1, were supplemented with* n*-3 fatty acids extracted from microalgae in lyophilized form and added to food; the experiment was carried out from week 8 to 16 of life. Flow cytometry was performed to determine the percentage of TCD4+ cells producing Th1 and Th2 cytokines.

**Results:**

Supplementation with microalgae fatty acids decreased the percentage of TCD4+ cells producing IFN-*γ* and TNF-*α* and increased the ones producing IL-17A and IL-12 in both strains; on the other hand, supplementation decreased percentage of TCD4+ cells producing IL-4 and increased the ones producing TGF-*β*.

**Conclusions:**

Microalgae* n-*3 fatty acids could be a useful tool in the treatment of diabetes as well as in the prevention of the appearance of health complications caused by inflammatory states.

## 1. Introduction

Diabetes mellitus is a multifactorial chronic noncommunicable disease, characterized by states of hyperglycemia resulting from defects in insulin secretion, its action, or both [1]. In Mexico, according to the National Health and Nutrition Survey Half Way 2016 (ENSANUT MC 2016 for its acronym in Spanish), 9.4% of the adult population has been diagnosed with diabetes [2]. It also represents the leading cause of negative health outcomes such as heart failure, blindness, kidney failure, amputations, and premature death [3]. The main cause of health complications in diabetes is chronic hyperglycemia, which is associated with changes in immunomodulation and inflammation [4].

The use of* n-*3 polyunsaturated fatty acids as a strategy to minimize damage caused by hyperglycemia has been deeply studied [5]. Its biological effects include benefits on the metabolism of lipoproteins [6], platelet, and endothelial and vascular function [7], as well as antioxidant and anti-inflammatory impact [8]. Evidence suggests that* n-*3 inhibits the proliferation of T lymphocytes in murine models and in humans [9, 10] and inhibits degranulation of cytotoxic T lymphocytes [11]. Thus, it suggests that polyunsaturated fatty acids have potentially immunosuppressive properties. Moreover, the supplementation with EPA (eicosapentaenoic acid) for 12 weeks has been shown to modify the fatty acids composition of the phospholipids of plasma, platelets, neutrophils, monocytes, and T and B lymphocytes [12].

Within their anti- and proinflammatory effects, it has been shown that, in cell cultures, EPA and DHA (docosahexaenoic acid) have high anti-inflammatory and immunosuppressive effects [13–15]. Same findings have been shown on animal studies supplemented with fish oil [16–18]. It has been proven that EPA and DHA supplementation decreases proinflammatory cytokines such as tumor necrosis factor Alpha (TNF-*α*), interleukin-6 (IL-6), monocyte-1 chemoattractant protein (MCP-1), and plasminogen activator-1 (PAI-1) inhibitor [19].

The main dietary sources of EPA and DHA fatty acids are fish, shellfish, and marine oils [20]. However, some disadvantages of the use of these sources are undesirable nutritional and organoleptic effects, such as oxidation (due to their high polyunsaturation) and the characteristic odor of the product [21]. Another disadvantage of the use of marine oils is the risk of contamination with heavy metals and pesticides, the solution of which may be oil refining, but this process involves a higher cost of production [22]. In addition, in recent years, marine sources have been diminished because fish catch has exceeded the maximum sustainable levels [23].

Microalgae are an evolutionarily microscopic diverse eukaryotic group of unicellular and predominantly aquatic photosynthetic organisms that have recently been studied for their potential to produce compounds of high biological value and beneficial for health such as carotenoids, polyunsaturated fatty acids, and very long chain polyunsaturated fatty acids [24]. They are the primary natural producers of EPA and DHA, because they have the biosynthetic machinery to sequentially alternate between desaturation and elongation in their carbon chains [25]. Because microalgae are at the beginning of the food chain, fish are the main consumers of these fatty acids, which is why they are incorporated into the lipids of membranes and accumulated in the fats and meat of many marine species [24]. However, there are fewer studies describing their anti-inflammatory effects as those already described for fatty acids of animal origin [26–28].

For all the above and the growing study of the composition and properties of microalgae, as well as the possibility of cultivating them in artificial form, interest has aroused in the study of these microorganisms as a renewable source of* n-*3 fatty acids. The aim of this study was to analyze the effect of the consumption of* n-*3 fatty acids extracted from microalgae (Chlorophyceae and Eustigmatophyceae families) either provided as a supplement or incorporated to diet, on the inflammatory markers from two different strains of mice: db/db mice as a model of obesity and diabetes mellitus in which an inflammation state is expected and CD1 mice as a model of optimal state of health without inflammation.

## 2. Methods

### 2.1. Animals and Study Groups

**Figure 1:**
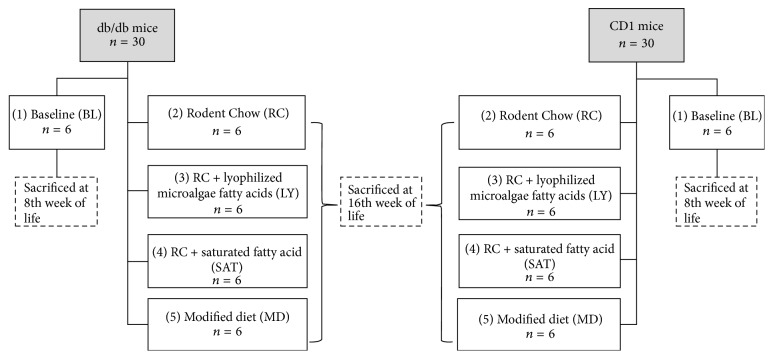
Experimental groups for both strains.

The present experimental, prospective, controlled, and randomized study was conducted with sixty 8-week-old male mice from two different strains: 30 db/db mice (BKS.Cg + Leprdb + LeprdbOlaHsd Harlan®) and 30 CD1 mice (Crl: CD1 (ICR) belonging to the Faculty of Medicine from the Autonomous University of Mexico State). For each strain, five study groups were formed (*n* = 6): (1) a baseline (BL) group to obtain baseline values; (2) a Rodent Chow (RC) group; (3) a RC + lyophilized microalgae* n-*3 fatty acids (LY) group; (4) a RC + saturated fatty acid (SAT) group; (5) modified diet (MD) supplemented with microalgae* n-*3 fatty acids group. Supplementation was administered from 8th to 16th week of life (Figure 1). The animals were housed in acrylic cages of 19 × 29 × 13 cm, with light/dark cycles of 12/12 h with controlled temperature at 21 ± 1°C. Groups 2, 3, and 4 were fed a standard normal diet (Rodent Laboratory Chow 5001 from Purina [3.02 kcal/g]) and water ad libitum. Water consumption (mL/week) and food (g/week) were recorded weekly. Animal care and experimental procedures in rodents were carried out in accordance with the rules of the Internal Regulations for the Use of Laboratory Animals and the Committee of Ethics in Research of the UAEMex, as well as the guidelines of the Ministry of Health and Agriculture of Mexico for the Production and Care of Laboratory Animals (NOM-062-ZOO-1999), Mexico City, Mexico. This protocol was approved by the Ethical Research Committee from the Faculty of Medicine of the UAEMex.

### 2.2. Obtaining of* n*-3 Fatty Acids (EPA and DHA) from Microalgae

The microalgae used in this project were native and collected and isolated by BIOMEX SA. de CV. The strains used were from Chlorophyceae and Eustigmatophyceae families which have a high content of EPA and DHA. The use of these microalgae for such purpose is of recent interest. The process for obtaining the EPA and DHA includes the cultivation of microalgae, separation of biomass, extraction of total lipids, and finally chromatographic procedures for EPA and DHA content determination (25.7% EPA + DHA). EPA and DHA were provided as free fatty acids form.

### 2.3. Supplementation

(a) LY group was fed with Rodent Chow and supplemented with lyophilized powder containing EPA + DHA obtained from microalgae. The supplemental dose was 1 mg/g of mouse weight, reconstituted in 100 *μ*l of distilled water, and administered with micropipette by direct oral deposition every day at 8:00 am.

(b) SAT group was fed with Rodent Chow and supplemented with coconut oil. The daily dose of coconut oil was 1 mg/g of mouse weight administered with micropipette by oral deposition at 8:00 am.

(c) MD group was fed with a Rodent Chow added with microalgae EPA + DHA for a total content of 2.0%* n-*3 fatty acid which means 10x the original content; Chow was administered ad libitum (Table 1).

### 2.4. Determination of Body Mass Index (BMI) and Blood Glucose Concentration

The BMI and blood glucose concentrations of animals were determined at the 8th and 16th week of life. The formula used for BMI determination was BMI = [weight (g)/length (cm)2 *∗* 100]. Weight was determined using a mouse Triple Beam 700/800 series Ohaus® brand weighing scale and length was determined by measuring the animal from nose to anus. Blood glucose was determined with a Bayer Contour TS glucometer through tail puncture.

### 2.5. Collection of Biological Samples

The BL groups were sacrificed at the 8th week of life and the rest of the groups were sacrificed at the 16th week of life. Animals were anesthetized by ether camera, bled by direct cardiac puncture (using a heparinized syringe, obtaining 1 mL of blood), and then sacrificed by cervical dislocation. 500 *μ*l of the collected blood was used for lymphocyte isolation using Ficoll-Hypaque Plus (GE Healthcare Bio-Sciences AB, Sweden); lymphocytes were stored with a PBS (phosphate-buffered saline) solution to obtain a final volume of 1 ml for further flow cytometry.

### 2.6. Flow Cytometry Assays

Cell suspensions of peripheral blood mononuclear cell (PBMC) were adjusted to 1 × 106 cells/mL in PBS for the cytofluorometric analysis with brief modifications [29]. (i) Surface phenotype of T cells was detected by using fluorescent labeled monoclonal antibodies: anti-CD3 FITC (Cat. number 553063), anti-CD8*α* PE (Cat. number 553035), and anti-CD4 PerCP (Cat. number 553052) (all from BD Biosciences). Cells were incubated for 30 min at room temperature. Finally, the cells were then washed with PBS and fixed in 1% paraformaldehyde. (ii) For the detection of intracellular cytokine production, lymphocytes were stimulated with a mixture containing phorbol myristate acetate, ionomycin, and Brefeldin A (Leucocyte Activation Cocktail Kit, BD Pharmingen) and incubated for 4 h at 37°C and 5% CO2. Then, antibodies to cell surface markers, anti-CD4 PerCP, were added and incubated as before. For intracellular staining of CD4+ T cells, fixation and permeabilization were performed using Cytofix/Cytoperm Kits (BD Pharmingen) according to the manufacturer's instructions. These cells were incubated with anti-IL-4 PE (Cat. number 554435), anti-IL-5 PE (Cat. number 554395), anti-IL-6 APC (Cat. number 561367), anti-IL-10 FITC (Cat. number 554466), anti-IL-17A FITC (BioLegend Cat. number 506907), anti-IFN-*γ* FITC (Cat. number 554411), and anti-TNF-*α* PE antibodies (Cat. number 554419). For all samples, the expression of CD69 was measured as an activation control. The fluorescent signal intensity was recorded and analyzed by FACS Aria Flow Cytometer (Becton Dickinson). Events were collected from the lymphocyte gate on the FSC/SSC dot plot. 20,000 gated events were acquired from each sample using the CellQuest research software (Becton Dickinson). Data was analyzed using Summit software v4.3 (Dako, Colorado Inc.). Data from six mice per group are reported as the mean ± standard deviation (SD).

### 2.7. Statistical Analysis

One-way ANOVA was performed for comparison between groups from each strain (BL, RC, LY, SAT, and MD); Bonferroni post hoc test was applied. Differences were considered significant at *p* < 0.05. Software used to run statistical analysis was SPSS v.23 for Windows.

## 3. Results

### 3.1. BMI Was Higher in the MD Group for Diabetic Mice and Blood Glucose Was Higher in All the db/db Groups

In the diabetic db/db mice, the MD group showed a significantly higher BMI than the BL group; blood glucose concentrations were significantly higher in all groups compared to the BL group; the food intake was significantly lower in the MD group and higher in the LY group and finally the water consumption was significantly higher in the MD group, all of this compared with the BL group (Bonferroni post hoc, *p* < 0.001). In the healthy mice (CD1), there were no significant differences in BMI and blood glucose. On the other hand, the consumption of food was significantly higher in the SAT group and the water consumption was significantly higher in the LY and SAT groups, all this compared with the BL group (Bonferroni post hoc, *p* < 0.001) (Table 2).

### 3.2. Microalgae Fatty Acids Modified Lymphocyte Populations in db/db Mice by Lowering CD3+ and CD8+ Populations and in CD1 Mice by Lowering CD3+

In the db/db strain, the percentage of CD3+ lymphocytes was significantly higher in all the groups when compared to the BL group (Bonferroni post hoc, *p* < 0.001). Regarding CD4+ lymphocytes, the MD group showed a significantly lower percentage and the SAT group a higher percentage (Bonferroni post hoc, *p* < 0.001). Finally, the CD8 + lymphocytes have a higher percentage in the MD, LY, and SAT groups (Bonferroni post hoc, *p* < 0.001).

For CD1 strain mice, the percentage of CD3+ lymphocytes was lower in the MD, LY, and SAT groups and higher in the RC group, all compared with the BL group (Bonferroni post hoc, *p* < 0.001). Regarding CD4 + lymphocytes, the percentage was significant in the MD and SAT groups (Bonferroni post hoc, *p* < 0.001). Finally, the percentage of CD8+ lymphocytes was significantly higher in the RC and LY groups, but lower in the MD and SAT groups, all compared with the BL group (Bonferroni post hoc, *p* < 0.001) (Table 3).

### 3.3. Supplementation with Microalgae Fatty Acids Increases IL-17A, IL-12, IL-4, IL-6, IL-10, and TGF-*β* but Decreases IFN-*γ*, TNF-*α*, and IL-5 in Diabetic Mice

In the diabetic mice, the behavior of Th1 type cytokines was the same for all study groups; the proportion of TCD4+ cells producing IFN-*γ* and TNF-*α* was significantly lower in all groups compared to the BL (Bonferroni post hoc, *p* < 0.001). On the contrary, the percentage of TCD4+ cells producing IL-12 and IL-17A was significantly lower in all groups (Bonferroni post hoc, *p* < 0.001). As for the Th2 type cytokines, the percentage of TCD4+ cells producing IL-4 was higher in the RC, MD, and SAT groups and, on the other hand, it was lower in the LY group. The percentage of TCD4+ cells producing IL-5 was significantly lower in all groups compared to the baseline group (Bonferroni post hoc, *p* < 0.001). For all groups, the percentage of TCD4+ cells producing IL-6 and IL-10 was significantly lower compared to the initial group (Bonferroni post hoc, *p* < 0.001). Finally, the percentage of TCD4+ cells producing TGF-*β* was significantly lower in the RC group and higher in the MD, LY, and SAT groups (Table 4).

### 3.4. Supplementation with Microalgae Fatty Acids Lyophilized Increases IL-17A but Decreases IFN-*γ*, TNF-*α*, IL-12, IL-4, and IL-6 in Healthy Mice

In CD1 mice, for Th1 type cytokines, the percentage of TCD4+ cells producing IFN-*γ* and TNF-*α* was significantly lower in the RC and LY groups and significantly higher in the SAT group (Bonferroni post hoc, *p* < 0.001). The percentage of TCD4+ cells producing IL-12 was significantly lower in the RC, MD, and LY groups and higher in the SAT group, all compared with the BL group (Bonferroni post hoc, *p* < 0.001); finally, the percentage of TCD4+ producing IL-17A was significantly lower in all groups compared to the BL group (Bonferroni post hoc, *p* < 0.001). Regarding the Th2 type cytokines, the SAT group showed a significantly higher percentage of TCD4+ cells producing IL-4, IL-5, IL-6, and TGF-*β* compared to the BL group (Bonferroni post hoc, *p* < 0.001), and the MD group showed similar behavior for IL-5 and TGF-*β*. On the other hand, the percentage of TCD4+ cells producing IL-4 and IL-6 was significantly lower in the LY groups (Bonferroni post hoc, *p* < 0.001) (Table 5).

## 4. Discussion

**Figure 2:**
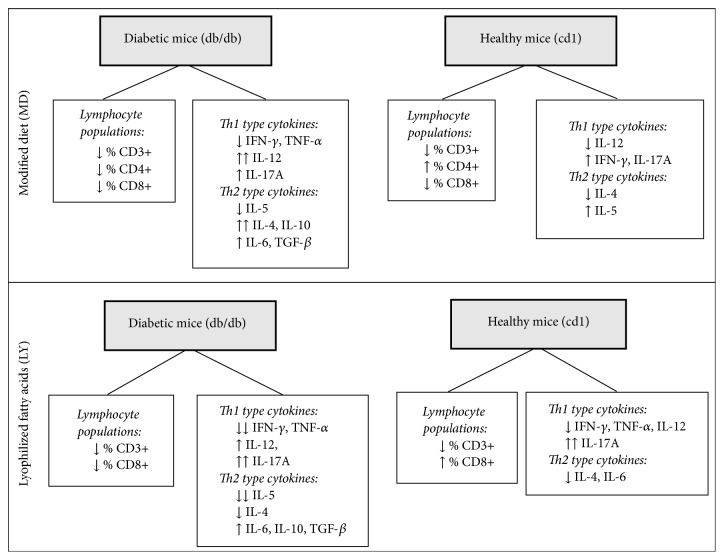
Summary of major findings about microalgae fatty acids supplementation in diabetic and healthy mice.

The results provided in this study show evidence that supplementation with* n-*3 fatty acids obtained from microalgae improves the inflammatory profile in general by reducing the secretion of many cytokines. Therefore, these results suggest that microalgae extracts may be considered as an anti-inflammatory strategy against different diseases. These findings are summarized in Figure 2.

The BMI was significantly higher in the MD group from the diabetic mice. These results are different from those reported by Zhuang in which C57B1/6 mice were supplemented with fish oil and did not show changes in BMI [30]. There were no significant differences in plasma glucose attributable to treatment; no studies were found to match our findings; however, a study with C57B/6 mice supplemented with EPA suggests a protective effect of* n-*3 fatty acids on glucose metabolism [19]. Further studies on the effect of microalgae fatty acids on glucose metabolism are needed.

In this study, food consumption was lower in the MD groups for the diabetic mice. Other studies with C57Bl/6 mice fed with normal chow enriched with EPA and DHA extracted from fish oil showed no changes in food consumption [31, 32]. However, a study from Díaz- Reséndiz explains that mice regulate food intake according to the composition of the food or the presence of an extra source of energy [33].

The percentage of total T lymphocytes was lower in all study groups from both strains. In contrast to this study, Marano et al. [34] suggest that consumption of* n-*3 fatty acids increases CD3+ lymphocyte populations including CD4+. In agreement with our findings, several supplementation studies report that there were no changes in lymphocyte populations [35, 36].

On the other hand, in both strains the SAT groups showed a significantly lower percentage of CD8+ cells. The CD4+ populations in the SAT groups increased significantly compared to their BL group. These results are consistent with those of Baccan et al. [37] who showed that consumption of high-fat diets significantly increases lymphocyte populations.

The db/db strain is characterized by a chronic inflammatory state such as diabetes disease, which causes the pro- and anti-inflammatory cytokines to be in higher concentrations compared to the CD1 strain; however, although the strains are very different between them, the decrease in cytokine concentrations occurred in a similar way.

In this study, supplementation with *n*-3 fatty acids extracted from microalgae significantly decreased the percentage of TCD4+ cells producing IFN-*γ* and TNF-*α*. These cytokines play different roles in inflammatory states such as in diabetes; IFN-*γ* directs the differentiation of CD4+ lymphocytes into helper lymphocytes type 1 (Th1); it also intervenes in the activation of macrophages and induces a greater secretion of IL-12 [38]. However, in the diabetic mice, microalgae fatty acids were shown to increase the percentage of TCD4+ cells producing IL-12. The main functions of IL-12 are the activation of Th1 lymphocytes and to stimulate the production of IFN-*γ* [39]. On the other hand, TNF-*α* is a cytokine involved in the acute and chronic phase as well as in the activation of the production of certain anti-inflammatory cytokines such as IL-4, IL-5, and IL-6 as a form of self-regulation of the inflammatory state [40].

A study by Vigerust et al. made in transgenic TNF-*α* C57B/6 mice that were fed with diets enriched with either fish oil or krill oil showed no modification in this cytokine [40]. Similarly, in a study on Wistar rats supplemented with fish or soybean oil, no significant differences were found in the concentrations of IFN-*γ* and TNF-*α* [41]. Although Xavier et al. [41] showed there are no changes in TNF-*α* after fish oil supplementation, there are also many studies that report a significant decrease in this cytokine [42–44]. A study made by Sierra et al. [45] reports that, in Balb/c mice fed with modified diet either with EPA or with DHA for 3 weeks, spleen lymphocytes decreased their production of TNF-*α* only in the diet with EPA, but not in the diet with DHA. When approaching the effect of microalgae fatty acids, a study carried out in cell lines of macrophages exposed to LPS and added with extracts of different microalgae showed a significant decrease of the TNF-*α* compared against the control cultures. A study by Sierra et al. reported that only EPA enriched diet was able to decrease IL-12 concentrations [45].

In this study, the percentage of TCD4+ cells producing IL-17A showed a significant increase in both strains. IL-17A is known as an inflammatory cytokine whose main function is exerted on myeloid and mesenchymal cells by inducing the expression of granulocyte colony-stimulating factor (G-CSF), IL-6, and other chemokines, which increase granulopoiesis and recruit neutrophils into the site of infection [46]. Vigerust et al. also reported that, after supplementation with fish oil and krill oil enriched diets, IL-17A was shown to be increased only in the fish oil group [40], and these results agree with our findings.

The supplementation with* n-*3 fatty acids in lyophilized form and that are added in the food showed a significantly higher percentage of TCD4+ cells producing IL-10 in both strains. IL-4 is produced by type 2 T cells (Th2), basophils, and mast cells. It has anti-inflammatory function by blocking the synthesis of IL-1, TNF-*α*, and IL-6. In addition, it promotes the proliferation and differentiation of B lymphocytes and is considered a potent inhibitor of apoptosis [47]. IL-10 is also known as cytokine synthesis inhibiting factor (CSIF) and can inhibit the synthesis of proinflammatory cytokines by T lymphocytes and macrophages. It also regulates the growth and differentiation of B lymphocytes, NK cytotoxic and helper T cells, mast cells, granulocytes, dendritic cells, keratinocytes, and endothelial cells [48]. A study in Wistar rats fed with a fish oil enriched diet was found to show lower concentrations of IL-4 and IL-5 at the alveolar level compared to control [41]. Also, Sierra et al. [45] showed similar results in Balb/c mice. Our findings agree with Li et al. [42] who demonstrated that fish oil supplementation decreased IL-10; however, Sierra et al. reported that only EPA supplementation was able to increase IL-10 concentrations [45]. These results suggest that the consumption of microalgae fatty acids has attenuating effects of systemic inflammation even in chronic diseases and not only in acute states of inflammation.

TGF-*β* is a cytokine with pleiotropic functions in hematopoiesis, angiogenesis, cell proliferation, differentiation, migration, and apoptosis. It has a strong anti-inflammatory action but may increase some immune functions. Thus, in knock-out mice for TGF-*β* they show defects in regulatory T lymphocytes, which generates an extensive inflammation with abundant proliferation of T lymphocytes and differentiation of CD4+ in Th1 and Th2 lymphocytes [49]. In our study both strains showed higher percentage of TCD4+ cells producing TGF-*β* in the LY group when compared with BL; however, they were much lower than those for the SAT group so we could agree that microalgae fatty acids have a positive effect on TGF-*β* expression. A study with apoE-deficient mice infused with angiotensin and treated with EPA and DHA orally for 3 weeks showed that the TGF-*β* gene expression was significantly decreased in the EPA group and DHA compared to untreated mice [50]. In contrast, a study on Wistar rats [51] exposed to LPS during gestation and whose offspring were supplemented with fish oil showed that TGF-*β* concentrations were increased as compared to controls [52].

IL-6 release is induced by IL-1 and TNF-*α*. It is involved in the production of immunoglobulins and in the differentiation of active B lymphocytes and plasma cells, modulates hematopoiesis, and is responsible, together with IL-1, for the synthesis of acute phase liver proteins like fibrinogen [53]. Percentage of TCD4+ cells producing IL-6 was shown to be significantly increased in LY group from both strains; however SAT group reported the highest IL-6 concentrations; we suggest that microalgae fatty acids could have a protective effect against IL-6 expression. Two studies report decreasing concentrations of IL-6 when using fish oil: one was conducted in mice [42] and the other in overweight pregnant women [43]. Additionally, a study by Robertson et al. [26] in macrophage cell line cultures treated with microalgae extracts showed a significant decrease in IL-6.

Regarding the effects of the consumption of coconut oil as a source of saturated fat, there is controversy about the properties of coconut oil, and this is because, despite being a source of saturated fat, several studies have shown that it has anti-inflammatory properties [54, 55]; in this study, the consumption of coconut oil showed a high percentage of TCD4+ cells producing Th1 and Th2 type cytokines which is consistent with other studies [56].

## 5. Conclusion

The results provided in this study show evidence that supplementation with* n-*3 fatty acids obtained from microalgae improves the inflammatory profile in general by reducing the secretion of many cytokines. Therefore, these results suggest that microalgae extracts may be considered as an anti-inflammatory strategy against different chronic diseases.

## Figures and Tables

**Table 1:** Nutrient composition of study groups' diet.

RC group		LY group		SAT group		MD group	
Protein, %	23.9	Protein, %	23.9	Protein, %	23.9	Protein, %	23.9
Starch, %	31.9	Starch, %	31.9	Starch, %	31.9	Starch, %	31.9
Glucose, %	0.22	Glucose, %	0.22	Glucose, %	0.22	Glucose, %	0.22
Fiber (crude), %	5.10	Fiber (crude), %	5.10	Fiber (crude), %	5.10	Fiber (crude), %	5.10
Cholesterol, ppm	200	Cholesterol, ppm	200	Cholesterol, ppm	200	Cholesterol, ppm	200
*EPA + DHA, %*	*0.2*	*EPA + DHA, %*	*0.2*	*EPA + DHA, %*	*0.2*	*EPA + DHA* ^*∗*^ *, %*	*2.0*
*Metabolizable energy:* *3.02 kcal/g*		*Metabolizable energy:* *3.02 kcal/g + 0.09 kcal/mg of lyophilized fatty acids*^*∗*^		*Metabolizable energy:* *3.02 kcal/g + 0.09 kcal/mg of coconut oil*		*Metabolizable energy:* *3.07 kcal/g*	

^*∗*^Microalgae source.

**Table 2:** Effect of supplementation with EPA and DHA extracted from microalgae on body mass index, blood glucose, food, and water consumption in db/db and CD1 mice.

	8 weeks old	16 weeks old			
	BL	RC	MD	LY	SAT
	Mean ± SD *n* = 6	Mean ± SD *n* = 6	Mean ± SD *n* = 6	Mean ± SD *n* = 6	Mean ± SD *n* = 6
db/db					
BMI g/cm^2^	57.3 ± 4.9	61.2 ± 2.5	65 ± 1.7^*∗*^	62.2 ± 1.5	59.7 ± 2.3
Glucose mg/dL	293.8 ± 131.0	551.8 ± 83.7^*∗*^	505 ± 74^*∗*^	525.5 ± 51^*∗*^	580.7 ± 22^*∗*^
Food intake, g/week	34.3 ± 2.1	32.9 ± 0.3	27.6 ± 1.0^*∗*^	37.5 ± 0.8^*∗*^	36.3 ± 2.4
Water intake, mL/week	64.8 ± 11.8	78.2 ± 7.2	81.4 ± 10.4^*∗*^	79.6 ± 1.8	67.2 ± 8.3
CD1					
BMI g/cm^2^	29.6 ± 2.0	31.4 ± 1.9	32.2 ± 3.1	34.4 ± 4.1	34.2 ± 2.4
Glucose mg/dL	126.2 ± 18.6	119.3 ± 13	127.5 ± 18.7	109.7 ± 13.3	106.8 ± 12.4
Food intake, g/week	54.6 ± 1.7	58.3 ± 5.6	42.7 ± 5	61 ± 1.9	73.3 ± 20.8^*∗*^
Water intake, mL/week	63 ± 13.1	67.3 ± 3.2	53.9 ± 1.3	89.8 ± 6.1^*∗*^	93.2 ± 4.8^*∗*^

Data are presented as means ± standard deviations. One-way ANOVA^*∗*^ for comparison of differences between BL group at 8 weeks versus all the groups at 16 weeks. *p* value was significant at <0.05.

**Table 3:** Effect of supplementation with EPA and DHA extracted from microalgae in lymphocytes populations in db/db and CD1 mice.

	8 weeks old	16 weeks old			
	BL	RC	MD	LY	SAT
	Mean ± SD *n* = 6	Mean ± SD *n* = 6	Mean ± SD *n* = 6	Mean ± SD *n* = 6	Mean ± SD *n* = 6
db/db					
CD3+, %	71.8 ± 0.5	70.4 ± 0.8^*∗*^	69.8 ± 0.4^*∗*^	70.7 ± 0.4^*∗*^	68.3 ± 0.2^*∗*^
CD4+, %	62.0 ± 0.2	62.1 ± 0.5	60.6 ± 0.3^*∗*^	61.8 ± 0.9	65 ± 0.5^*∗*^
CD8+, %	30.0 ± 1.0	29.3 ± 1.0	24.9 ± 0.3^*∗*^	28.4 ± 0.2^*∗*^	23 ± 0.1^*∗*^
cd1					
CD3+, %	74.7 ± 0.02	77.8 ± 0.04^*∗*^	70.6 ± 0.03^*∗*^	72.4 ± 0.01^*∗*^	69.5 ± 0.06^*∗*^
CD4+, %	67.4 ± 0.2	67.5 ± 0.05	70 ± 0.04^*∗*^	67.6 ± 0.03	72.3 ± 0.4^*∗*^
CD8+, %	21.4 ± 0.05	24.8 ± 0.01^*∗*^	17.4 ± 0.1^*∗*^	24.2 ± 0.03^*∗*^	19.9 ± 0.02^*∗*^

Data are presented as means ± standard deviations. One-way ANOVA^*∗*^ for comparison of differences between BL group at 8 weeks versus all the groups at 16 weeks. *p* value was significant at <0.05.

**Table 4:** Effect of supplementation with EPA and DHA fatty acids extracted from microalgae on Th1 and Th2 cytokines in db/db.

db/db mice	8 weeks old	16 weeks old			
	BL	RC	MD	LY	SAT
	Mean ± SD *n* = 6	Mean ± SD *n* = 6	Mean ± SD *n* = 6	Mean ± SD *n* = 6	Mean ± SD *n* = 6
Th1 % TCD4+/					
IFN-*γ*	22.5 ± 0.4	11.5 ± 0.4^*∗*^	7.2 ± 0.5^*∗*^	2.1 ± 0.4^*∗*^	15.7 ± 0.4^*∗*^
TNF-*α*	10.2 ± 0.4	7.7 ± 0.4^*∗*^	8.8 ± 0.4^*∗*^	2.4 ± 0.4^*∗*^	1.4 ± 0.4^*∗*^
IL-12	1.2 ± 0.1	12.4 ± 0.5^*∗*^	10.2 ± 0.5^*∗*^	6.8 ± 0.1^*∗*^	3.6 ± 0.5^*∗*^
IL-17A	1.4 ± 0.1	4.2 ± 0.3^*∗*^	3.7 ± 0.1^*∗*^	11.4 ± 0.3^*∗*^	11.4 ± 0.3^*∗*^
Th2 % TCD4+/					
IL-4	1.8 ± 0.08	8.2 ± 0.3^*∗*^	6.6 ± 0.3^*∗*^	1.1 ± 0.1^*∗*^	10.3 ± 0.2^*∗*^
IL-5	19.9 ± 0.8	9.4 ± 0.4^*∗*^	7.5 ± 0.6^*∗*^	2.1 ± 0.2^*∗*^	6.5 ± 0.2^*∗*^
IL-6	1.5 ± 0.1	3.1 ± 0.4^*∗*^	4.5 ± 0.2^*∗*^	4.0 ± 0.1^*∗*^	14.8 ± 0.2^*∗*^
IL-10	1.44 ± 0.2	16.0 ± 1.3^*∗*^	7.3 ± 0.2^*∗*^	2.7 ± 0.08^*∗*^	14.5 ± 0.2^*∗*^
TGF-*β*	3.2 ± 0.4	2.1 ± 0.05^*∗*^	5.5 ± 0.1^*∗*^	4.9 ± 0.09^*∗*^	7.1 ± 0.1^*∗*^

Data are presented as means ± standard deviations of percentage of TCD4+ cells producing cytokines. One-way ANOVA^*∗*^ for comparison of differences between BL group at 8 weeks versus all the groups at 16 weeks. *p* value was significant at <0.05.

**Table 5:** Effect of supplementation with EPA and DHA fatty acids extracted from microalgae on Th1 and Th2 cytokines in CD1.

CD1 mice	8 weeks old	16 weeks old			
	BL	RC	MD	LY	SAT
	Mean ± SD *n* = 6	Mean ± SD *n* = 6	Mean ± SD *n* = 6	Mean ± SD *n* = 6	Mean ± SD *n* = 6
Th1 % TCD4+/					
IFN-*γ*	1.8 ± 0.18	1.2 ± 0.09^*∗*^	2.7 ± 0.1^*∗*^	1.0 ± 0.1^*∗*^	4.3 ± 0.1^*∗*^
TNF-*α*	2.5 ± 0.13	1.5 ± 0.1^*∗*^	2.6 ± 0.1	1.4 ± 0.09^*∗*^	4.6 ± 0.07^*∗*^
IL-12	2.8 ± 0.1	1.7 ± 0.08^*∗*^	2.4 ± 0.09^*∗*^	1.3 ± 0.08^*∗*^	4.4 ± 0.08^*∗*^
IL-17A	1.6 ± 0.08	1.8 ± 0.08^*∗*^	1.8 ± 0.1^*∗*^	4.1 ± 0.08^*∗*^	3.6 ± 0.09^*∗*^
Th2 % TCD4+/					
IL-4	2.6 ± 0.1	1.8 ± 0.09^*∗*^	2.4 ± 0.1	1.6 ± 0.1^*∗*^	4.4 ± 0.1^*∗*^
IL-5	2.1 ± 0.1	1.4 ± 0.1^*∗*^	2.6 ± 0.1^*∗*^	1.9 ± 0.1	3.4 ± 0.1^*∗*^
IL-6	1.8 ± 0.1	1.7 ± 0.08	1.7 ± 0.1	1.5 ± 0.09^*∗*^	2.6 ± 0.07^*∗*^
IL-10	1.6 ± 0.08	1.5 ± 0.1	2.2 ± 0.1	1.2 ± 0.1	2.9 ± 1.6
TGF-*β*	1.5 ± 0.06	1.3 ± 0.8^*∗*^	2.3 ± 0.07^*∗*^	1.5 ± 0.08	5.1 ± 0.1^*∗*^

Data are presented as means ± standard deviations of percentage of TCD4+ cells producing cytokines. One-way ANOVA^*∗*^ for comparison of differences between BL group at 8 weeks versus all the groups at 16 weeks. *p* value was significant at <0.05.
